# An Evaluation of Telepractice use During the Covid-19 Pandemic for the Treatment of Speech and Language Disorders in Belgium

**DOI:** 10.5195/ijt.2022.6411

**Published:** 2022-06-03

**Authors:** Ronny Boey, Stefaan Lefevere

**Affiliations:** 1 University of Antwerp, Faculty of Medicine and Health Sciences, Antwerp, Belgium; 2 Study Department VVL, Belgium

**Keywords:** COVID-19 pandemic, Speech-language pathology, Survey, Telepractice

## Abstract

The aim of this article was to evaluate the sudden implementation of telepractice in Belgium during the COVID-19 pandemic. A 38-question survey was completed by 1,222 Dutch-speaking speech-language pathologists (SLPs) from Belgium. Most reported good or very good satisfaction with telepractice and that telepractice can be effectively used with clients of different ages and speech disorders with or without comorbidity. The SLPs reported when telepractice could be used most effectively. They also detailed their difficulties with both technology and client-related issues. Limitations when switching to telepractice included a lack of training and experience, and digital materials.

The main objective of this study was to define telepractice in Belgium during the pandemic. A second aim was to compare the findings with those from similar studies to identify the advantages and disadvantages of telepractice and thus support decisions on how to continue telepractice in the near future.

The American Speech-Language-Hearing Association (ASHA, 2022b) defines telepractice as “the application of telecommunications technology for delivery of professional services at a distance by linking clinician to client, or clinician to clinician, for assessment, intervention and/or consultation” (para. 1). ASHA suggests use of the terms teleaudiology, telespeech, and the over-arching term, telepractice.

Telemedicine, and by extension telehealth for other health professions, can according to the Commission of the European Parliament (2022), be defined as:

the provision of health services by means of ICT in situations where the health professional and the client (or two health professionals) are not in the same place. It involves the secure transmission of medical data and information in the form of text, sound, images, or other forms necessary for the prevention, diagnosis, treatment, and follow-up of clients.

At the end of 2019, the virus SARS-CoV-2 or COVID-19 emerged in China and developed into a pandemic within a few months. In many countries and regions, the cessation of speech-language therapy and the prohibition of non-essential travel were imposed for several months in 2020.

In Belgium, the National Institute for Health and Disability Insurance (NIHDI) allowed telepractice for the first time ever on 14 March 2020 to ensure the continuity of speech and language services during the COVID-19 crisis. To continue the telepractice services during the pandemic, the NIHDI required feedback on the use of telepractice by SLPs. NIHDI emphasized the importance of continuing care for the clients of SLPs. This position was also stressed by pediatricians (Tohidast et al., 2020).

Three articles employed survey methodology to examine the use of telepractice and informed this research. Two surveyed SLPs (Fong et al., 2021; Vrinda & Reni, 2020) and one surveyed clients (Tenforde, et al., 2020). The studies are summarized below.

## SPEECH LANUGAGE PATHOLOGISTS IN HONG KONG

Fong et al. (2021) surveyed 135 SLPs with an average of 8.11 years of experience (*SD* = 7.12 years) on the use of telepractice during the COVID-19 period in Hong Kong. The SLPs worked in multiple settings: kindergarten, primary school, and special education (65.9%); clinic (18.5%); private practice (18.5%); nursing home (16.3%) or university (7.4%). Clients had articulation disorders (83%), language development disorders (77.0%), social communication disorders (62.2%), motor speech disorders (58.5%), and voice disorders (53.3%). Dysphagia or eating disorders (48.9%) and stuttering were slightly less common (43%), followed by aphasia (38.5%), multimodal communication (31.1%), hearing loss (30.4%), and dyslexia (23.0%).

Of the 135 SLPs, most (72.3%) offered telepractice for less than 3 months. Approximately one-third of the SLPs (34.0%) used telepractice for screening (17.0%) or diagnostics (15%). A wide age range of clients received services: toddlers (< 2 years: 12.8%), preschoolers (3-5 years: 34%), children and teenagers (6-17 years: 42.6%), adults (18-40 years: 25.6%), and older adults (41-64 years: 31/9%; ≥ 65 years: 29.8%).

Video-conferencing technology was used for the majority of clients (70.2%). The chief concerns of respondents were technological issues with hardware and software, the stability of the internet connection, and the lack of training. The most common reason (83.0%) for not using telepractice was the type and age of clients. Other reasons included: that telepractice was impersonal and prevented physical contact (52.2%), technological barriers (50.0%), a perception of being ineffective (40.9%), refusal by the care provider or client (33.0%), no apparent need to change the intervention system (25.0%), ethical aspects (13.6%), and higher costs (5.7%). Forty-seven respondents (51.1%) indicated that therapy via telepractice was less effective than in-person treatment, while 25.5% experienced telepractice as equally effective. No respondent found telepractice more effective than in-person therapy.

## SPEECH LANGUAGE PATHOLOGISTS IN KERALA REGION, SOUTHWEST INDIA

Vrinda and Reni (2020) evaluated telepractice during the COVID-19 pandemic by surveying 105 SLPs from the Kerala region (Southwest India); 104 SLPs completed the questionnaire. The survey (presented via an online Google form) contained 25 questions, 22 of which had a closed response pattern. The SLPs' professional experience ranged from 6 months to 30 years. The work setting was academic for 33.7% and a clinical setting for 45.3%. Special education was the work setting for 2.9%, a rehabilitation center for 5.8%, and a few other settings for < 1.9% of the SLPs surveyed. While (74%) used telepractice during the COVID-19 period, 79.2% had no experience with telepractice before the pandemic. These SLPs reported to have prepared themselves by watching webinars (61%), reading articles (49.4%), acquiring skills through trial and error (71.4%), discussing telepractice among colleagues (71.4%), training parents (1.3%), consulting YouTube on telerehabilitation (1.3%), consulting the ethical code for speech and audiology, and verifying the protection of clients' privacy. Telepractice was used for counselling and guidance by most clinicians (both 87%), for follow-up (70%), diagnosis/evaluation (63.6%), and screening (32.5%). The ages of the clients ranged from younger than 6 months (3.9%) to older than 60 years (10.4%). The mode was between 3.5 and 11 years (70.1%).

The most commonly treated disorders were language disorders (53.2%), autism spectrum disorders (46.8%), articulation disorders (41.6%), stuttering (39%), learning disabilities and hearing loss (both 24.7%), aphasia (23.4%), and general developmental delay (23.4%). Other disorders less reported for telepractice use included voice and resonance disorders (15.6%), ADHD (15.6%), dysarthria (14.4%), cerebral palsy (13%), cognitive communication disorders (10.4%), auditory verbal therapy (10.4%), alternative augmentative communication (9.1%), dysphagia (1.3%), and verbal apraxia (1.3%). Children with ADHD and with autism spectrum disorders were perceived as the most difficult group to treat with telepractice. Dysphagia was rarely treated, probably because of the perceived risks involved in telepractice, although guidelines exist on how to do so safely, feasibly, and reliably (Georges, et al., 2006; Miles, et al., 2020).

The sudden need to switch to telepractice raised several issues. It was difficult to treat children through telepractice (76.6%), there was a lack of online tools (46.8%), and some clients were not willing to receive online care (44.2%). Regarding technical issues, 94.9% of participants reported problems with internet connections.

Advantages were perceived in the use of telepractice during the COVID-19 period. Many SLPs (54.5%), reported more regularity in client attendance, with client contact increasing (53.2%). Not having to travel with the risk of contracting the COVID-19 virus was mentioned by 92.2% as an advantage of telepractice. According to 61%, parents could be more involved in the therapy. In addition, 84.5% of the SLPs reported that with telepractice, clients felt more comfortable carrying-out activities from home. A further advantage was that follow-up and supervision with telepractice was perceived as easy by 46.8% of the SLPs. Client satisfaction with telepractice was perceived as “very good” by 9.1%, and as “good” by 50.6%, with 39% reportedly “satisfied” and only 1.3% “not satisfied.”

## CLIENTS IN MASSACHUSETTS, USA

Tenforde et al. (2020) conducted a survey of the application of telepractice during the COVID-19 period in Massachusetts (USA). They assessed its feasibility and satisfaction in a multidisciplinary context (i.e., physical therapy, occupational therapy, and speech therapy). Of 211 clients surveyed, 205 completed a questionnaire. Of the 205 clients, 110 identified as female (53.7%), 92 as male (44.9%) and 3 as transgender (1.5%). A quarter (25.4%) were 7 years old or younger. The second largest group (32.7%) was aged between 35 and 64 years. About one fifth (19.5%) were 64 years of age or older.

The online survey contained 16 multiple-choice questions answered with a choice of: “weak,” “sufficient,” “good,” “very good,” and “excellent.” Client-related data included: gender, age (0 to ≥ 65 years), insurability, travelling time without telepractice, support in the environment, type of therapy, whether the treatment was new or continued, the duration of treatment, and the diagnosis or condition. The latter included neurogenic communication disorders (e.g., post stroke, concussion, post-traumatic brain injury, Parkinson, and pediatric neurologic conditions).

Clients were asked about the quality aspects of telepractice, including how the communication with the therapist was experienced, how the therapy was perceived, and convenience. Since all clients were insured for the care they received, financial charges did not prevent them from participating in telepractice. The mode for time spent in a session was between 30 and 45 minutes for 59.5% of the clients.

Clients were also asked about available help from friends or family. Of the respondents, 52.2% reported that they could not call on help with telepractice from a friend or family member, 39.0% indicated that they could rely on their help with telepractice, and 8.8% said they could call on remote assistance.

The clients were asked about their experience with telepractice services. Overall the answer “excellent” varied between 70% and 90% depending on the aspect of telepractice queried. In addition, 10-20% reported “very good” and 3-10% answered “good.” There was no correlation between the age of a client and the evaluation. Female clients reported a slightly higher level of satisfaction.

## METHODOLOGY

### STUDY APPROVAL

The procedure of developing the survey in the current study, inviting SLP members of VVL, and analyzing the results was reviewed and approved by an independent scientific committee Scienti-L with members of the University of Antwerp, the University of Ghent, and the University of Louvain.

### DEVELOPING THE SURVEY

To develop the survey questions, the findings of a systematic review of studies with telepractice served as a source of information (Boey, 2014, 2015) as well as the three studies on pandemic-based telepractice described above. The survey construction was also informed via collaboration with a sociologist and a statistician with professional experience in survey development and the online software used. We chose CheckMarket® as an online tool in function of the ease of use for the respondents, for the processing and display of the results, for the security of data storing in datacenters, and use of two-step verification. Before release, the survey was pilot tested by three SLPs.

In total, 38 questions were asked. These are listed in the Appendix. Questions about diagnostics, screening, and testing were not included as they were not allowed by NIHDI. The questions explored a number of aspects of telepractice: (a) the reasons to use or not use telepractice (questions 1-5), (b) the frequency of use of telepractice versus in-person treatment (questions 6-10), (c) the treated disorders and setting (questions 11-16), (d) the use of technology and possible issues (questions 17-20), (e) the feasibility and accessibility (questions 29-34), (f) the potential future use of telepractice (questions 35-37) and (f) additional remarks and comments. The answers included. “yes-no,” “yes rather often,” “yes occasionally,” “no,” “very dissatisfied,” “rather dissatisfied,” “rather satisfied,” “very satisfied.” Some multiple-choice answers allowed descriptive choices (e.g., about internet connection, sound and vision, digital display of material, interactions between SLPs and client, and privacy). A number of questions allowed for open answers.

### TIMING

The survey was launched in September 2020, allowing the researchers to examine experiences after telepractice launched in Belgium (i.e., predominantly in April 2020). During that time, SLPs suddenly had to master telepractice through training, become familiar with video conference technology, provide interactive material digitally, and draw up recommendations for clients.

#### INVITATION TO PARTICIPATE IN THE SURVEY

The Vlaamse Vereniging voor Logopedisten (i.e. the Flemish Association of Speech-language therapists, acronym: VVL) has access to the largest group of SLPs in Belgium practicing in various settings. Moreover, each individual in this group could be reached by e-mail to invite them to participate in the survey. That is why these 2,699 SLPs were invited to participate in the survey. The invitation was sent to each member by means of an eNewsletter of VVL with a link to the survey. A reminder was sent ten days later to those who had not yet participated. The survey ran for 18 days. Participation was anonymous. The e-mail addresses used for the invitation were sent by Campaign Monitor® and segregated from the CheckMarket online-tool so that no names or other identifying information were requested, registered, or used in the processing of results. Of the 2,669 invitations sent, only four bounced back (0.1%).

#### PROCESSING AND ANALYZING THE RESULTS

The CheckMarket® application allowed reports to be generated automatically, including percentage numbers of responses, graphs (bar charts, pie charts), and qualitative responses that could be classified by frequently used key words. The VVL Study Department processed the results in consultation with the sociologist and statistician. Numerical and percentage and central statistical tendencies figures were calculated.

## RESULTS

### SURVEY RESPONDENTS

A total of 1,222 members responded to the survey, a response rate of 45.3%. Of the respondents, 194 completed the survey partially (15.9%) and 1,028 (84.1%) completely. Only 171 (14%) indicated that they did not use telepractice. Most respondents were female (97.6%) and the average age was 33 years 8 months (*SD* = 10).

### CLIENT AGES

The youngest person to be treated via telepractice was 3 years10 months and the oldest was 72 years old. Almost every SLP (96%) reported having engaged in telepractice with children between the ages of 7 and 12 years. SLPs provided service via telepractice to adults (33%), to teenagers between the ages of 13 and 17 years (61%), and to toddlers between 2 and 6 years of age (43%). Parent counselling was provided to 15%. This wide age range reflects the accessibility of the health care system and reimbursement in Belgium.

### INTENSITY OF THE USE OF TELEPRACTICE

The NIHDI-regulations required a duration of 30 minutes for an online therapy session. The average number of online sessions of 30 minutes weekly in the month before the survey was less than 10 for 41% of the SLPs and between 11 and 20 for 34% of the SLPs.

### SPEECH AND LANGUAGE DISORDERS

Dyslexia, dyscalculia, language development disorders, articulation disorders and oro-myofunctional disorders were treated more by telepractice than in-person therapy. This was reported by over half of the participants. About a quarter reported online therapy for aphasia, voice disorders, and dysarthria. Almost a fifth treated dysphagia and dyspraxia. Stuttering (12%), cluttering (6%), and hearing loss (5%) were less frequently treated online. Most SLPs (93.5%) indicated that when in-person therapy with COVID-protective measures became possible, they reduced the number of telepractice sessions as compared to the lock-down period. The three main reasons were: the choice of the client or parents (37.6%), the contact between therapist and client, the greater flexibility of the interaction, and the availability of more materials (26.9%).

### CO-MORBIDITY

The registration of existing co-morbidity is part of the initial diagnosis of a SLP in cooperation with a physician. A total of 933 SLPs reported the extent to which in-person or telepractice was used as a treatment modality when a co-morbid disorder such as Attention Deficit Hyperactivity Disorder (ADHD), Attention Deficit Disorder (ADD) or Autism Spectrum Disorder (ASD) was present. For all specified co-morbid disorders, in-person treatment was more frequently used than telepractice.

### CONDITIONS FOR TELEPRACTICE USAGE

Three types of conditions influenced telepractice usage. First, were conditions related to a properly functioning computer (i.e., sound, camera, microphone, headset), software (i.e., video conferencing software, specific software for telepractice and digital materials) and the Internet connection (i.e., stable and fast enough). These accounted for 50.0% of the responses.

A second consideration accounted for 23.5% of the response. This related to clinician effort and time investment. The SLPs' reported that their time investment was much higher for telepractice than for in-person therapy.

A third consideration, accounting for 26.3% of the responses, related to client factors, such as being able to work with the computer at a distance, (which sometimes implied disorder-related exclusion), having the motivation or interest to do so, and being able to realize the tele-interaction with comfort and privacy.

### SETTINGS

SLP respondents practiced in schools (35.6%), rehabilitation centers (60.0%), special education (35.7%), hospitals (7.8%) or elsewhere (6%) (e.g., a care home) or in a combination of settings.

### TECHNOLOGY

#### HARDWARE AND SOFTWARE

A small proportion of respondents indicated use of more than one system. The majority used a portable computer (71.5%), with less using a desktop computer (15.3%), a tablet (7.6%) or a smartphone (5.6%). For more than half, Zoom (55%) was used as video technology software.

#### TECHNICAL ACCESSIBILITY

For most SLPs (84%) the technical access to telepractice occurred with few or no problems. Eight reported problems with creating or using the link. Operating the software caused problems for a small proportion of respondents (6%).

Technical accessibility was easy for 69% of the clients. Approximately one in five clients (21%) had problems operating the software and 21% had problems using the link. Difficulties logging in were reported for 18% of the clients; and 7% had difficulties turning on the camera or microphone.

Overall, 44% of the SLPs reported a technical problem with telepractice, occurring occasionally (26%) or sometimes (27%). Problems with the internet connection were reported most often by the SLPs (86%). In addition, there were problems with sound (e.g., distortion, reverberation) for 50% of respondents. Problems with visual acuity (e.g., image, lighting) were reported by 23%.

### EFFORT, TIME, AND COST

#### PREPARING FOR THERAPY WITH TELEPRACTICE

Preparing for therapy with telepractice took more time than the in-person speech therapy, according to 85% of the respondents. For 13% there was no difference and for 2% preparation took less time. To improve their use of telepractice the SLPs put extra time and effort in education to learn how to work with the software (78%). Many SLPs informed themselves about the use of telepractice (62%). This occurred via a brief training course (41%), searching for digital materials for telepractice made available by VVL in a DIGICENTER (28%), and obtaining technological tools (6%).

#### TELETHERAPY CLINICIAN EFFORT

Prior to the COVID19 pandemic, speech therapy sessions were held “back-to-back” without the need for between session preparation delays. In contrast, during the COVID-19 pandemic fewer clients could be seen each day because of sanitation between sessions, lag-time between clients, and time connecting to tele-sessions. This was reported by 63% of the respondents. It is therefore not surprising that a majority of respondents (83%) indicated that more effort was required to provide telepractice compared with in-person therapy. For 15% of respondents, the effort was similar and for 2% it was less.

#### COSTS

While 52% of the SLPs reported that the cost of telepractice was the same as the cost of in-person therapy, 28% reported that the cost of telepractice was higher than for in-person therapy, and 20% replied it was less.

#### SLP AND CLIENT SATISFACTION

**Figure 1:**
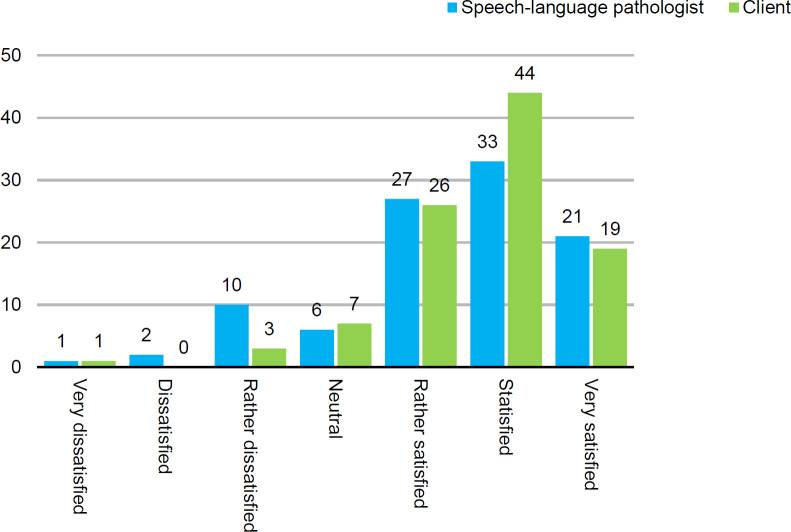
SLP Responses (Percentages) on Satisfaction with Telepractice for both Themselves and for their Clients

The SLP participants were asked to indicate the degree of overall satisfaction for both themselves and for their clients using a 7-point scale (i.e., “very dissatisfied,” “dissatisfied,” “rather dissatisfied,” “neutral,” “rather satisfied,” “satisfied,” and “very satisfied”). The distribution of the answers given to both questions is shown in Figure 1. It is clear the distribution is skewed to the right, with a much higher degree of satisfaction with telepractice reported.

### ADVANTAGES AND DISADVANTAGES OF TELEPRACTICE FOR CLIENTS

The participating SLPs were asked to mention the advantages and disadvantages perceived by their clients. Eighty SLPs listed 247 advantages (Table 1) and 73 SLPs reported 156 disadvantages (Table 2). In order of importance, 40.1% reported the advantage of not needing to travel to the speech therapy practice, 23.5% reported that telepractice enabled continuity of treatment, and 12.1% reported an increase of motivation with telepractice. Other advantages were less frequently reported.

**Table 1:** Advantages of Telepractice (Reported by SLPs as Perceived by Clients)

Advantage	Description	Percentage (%) of total respondents (247)
**Conditions**		
Displacement	Time gain, less effort, less cost	40.1
At home	Convenience, familiar environment, partner, or parent can follow along	2.0
**Therapy**		
Continuity	Continuous care is possible, no interruption	23.5
Safety	No risk of infection, possibly in quarantine, for clients at risk	8.9
Motivation	Working independently with computer motivates, new style, exercises	12.1
Content	Flexibility, individual adjustment, variation of exercises	5.7
Efficiency	Regularity, effects, transfer of practice material	3.2
Parents	Present, more involved, cooperative	4.5

Table 2 shows the disadvantages reported by SLPs as perceived by clients (Appendix, Question 4). Missing in-person interaction with the SLP was cited by 18.6%. The unavailability of a computer (i.e., due to no available computer, or that other children or parents needed to use the computer) and an unstable or insufficiently fast internet connection were each reported in 16% of the responses. Not being able to implement specific interventions via telepractice constituted 15.4% of the responses. Other disadvantages were less frequently reported.

**Table 2:** Disadvantages of Telepractice (Reported by SLPs as Perceived by Clients)

Disadvantage	Description	Percentage of total answers (156)
**Technology**		
Computer	Availability and equipment (camera, sound)	16.0
Internet	Unstable, insufficient speed	16.0
Software	Problems with the operation of the videoconferencing software	10.3
**Therapy conditions**		
Attention	Insufficient concentration, distraction	13.5
Conditions	Unsettled home situation	5.8
Client-therapist interaction	Insufficient interaction, feeling of lack of human contact	18.6
Content	Certain interventions were perceived as not possible (e.g., tongue strength training, manipulation, treatment of dysorthography)	15.4
Disorder	Perceived as not possible with paresis, hearing impairment, mental disability, or oro-myofunctional training	4.5

### ADVANTAGES AND DISADVANTAGES OF TELEPRACTICE FOR SLPS

The four most frequently mentioned advantages reported (Table 3, and Appendix, Question 25) were: (a) the safety of telepractice with no risk of contamination by COVID-19, (b) telepractice was a good alternative to therapy in the lock-down situation, (c) telepractice led to more structured therapy, and (d) telepractice required no travel for the therapist. Other less frequently stated advantages are shown in Table 3.

**Table 3:** Benefits of Telepractice Reported by SLPs

Benefits	Percentage (%) compared to total answers (283)
More structure in speech therapy	17.0
Safer in terms of health (colds, infections, etc.)	19.4
Alternative if the practice is not allowed to open (financial, continuity)	18.0
More parental involvement	7.8
More insight into the client's personal situation	1.1
SLPs can work with material from the client	0.7
No more travel for SLP (e.g., for home visits) and client	12.0
There are no advantages	2.1
Questions and sharpens creativity	4.9
Increased client motivation	2.5
SLPs can serve clients who are on holiday or with grandparents	3.9
Fewer cancellations	4.2
Digital material can be easily shared	1.1
SLPs can stop and start sessions with more punctually	1.4
Useful but only as a supplement to / alternation with physical sessions	3.9

Disadvantages of telepractice were reported by 142 respondents for a total of 437 comments (Table 4, and Appendix, Question 26). The four most frequent reported disadvantages were: (a) the need of more time to prepare a therapy session, (b) less flexibility to use or switch material in the online therapy session, (c) more stress due to focusing on the computer screen, and (d) less possible interaction between therapist and client. Other disadvantages were related to technical issues, environmental, and specific therapy elements.

**Table 4:** Percentage of Reported Disadvantages of Telepractice Completed by 142 SLP Respondents and 437 Answers (Question 26)

Description	Percentage (%) compared to total answers (437)
More preparation time needed	15.6
Less interaction between client and therapist	11.2
Less efficient/impossible with certain disorders/people/types of therapy	9.8
Less flexible in use of material/in variation of therapy/adjustment of therapy	11.9
SLPs has less control over the child, less feeling with the child.	8.9
Negative effect on child concentration/distraction within home environment	6.4
Instructions do not come across as well	2.7
Field of vision on the child is more limited (e.g., breathing, body language)	3.2
Very tiring/stressful due to constant focus on the screen	11.7
Play therapy is more difficult	2.1
Computer skills are less in some clients	3.7
Stramien on the voice	1.4
Unstable internet connection/sound	7.3
Vulnerable families have only smartphone available	3.0
Therapy sessions follow each other less quickly	1.1

### FUTURE APPLICATIONS

To the question of whether speech-language pathologists will work with telepractice in the future, almost half (48%) replied they certainly would. For this group, it is important to note that the application possibilities were most developed. Only 9% indicated that they will definitely not use telepractice in the future.

The group of clients for whom telepractice will be offered in the future was perceived as diverse. Children (7-12 years) and teenagers (13-18 years) represented the largest part (reported by 89% and 80% of respondents, respectively). Nearly half of the respondents (48%) indicated future use of telepractice for interdisciplinary consultation (i.e., with teachers), and 37% for parental guidance. Of the SLPs surveyed, 87% expect to use telepractice with clients with dyslexia or dyscalculia. Less frequently reported was the future use of telepractice for the treatment of dysortography (41%), dysphasia (41%), articulation disorders (26%), voice disorders (21%), dysarthria (13%), oro-myofunctional disorders (11%), aphasia (11%), stuttering (9%), cluttering (3%), hearing disorders (2%), dysphagia (1%), and others (5%). These results most likely reflect the prevalence of a disorder, the extent to which it is represented in a practice, and the reimbursement provided by NIHDI.

### COMMENTS, SUGGESTIONS, CONCERNS

As a final question (Appendix, Question 38), respondents were given the opportunity to provide additional comments, suggestions, and reservations. They expressed the following:

- The wish to keep telepractice possible in the future after the COVID-19 period as a supplement to in-person speech therapy and as an alternative, chiefly due to problems with travelling due to traffic or restricted mobility of a client (expressed by 43.5%).
- That telepractice can be used for individual treatment if the client meets certain selection criteria. This should be considered for each individual client (expressed by 28.9%).
- Support for further development of possibilities with telepractice is welcome (e.g., digital material, manual, training, education) (expressed by 11.8%).
- The effort required to prepare and provide telepractice is higher than for the in-person situation (expressed by 11.8%).

## DISCUSSION

This study evaluated the sudden and first-time usage of SLP provided telepractice in Belgium during the first wave of the COVID-19 pandemic. During that period, the NIHDI mandated that only telepractice could only be used to continue, not initiate, speech therapy.

Reported data on the use of telepractice was obtained from 1,028 SLPs who fully answered an online survey with 38 questions. The data indicated that telepractice has been widely used with clients of a range of ages (3 to 72 years old) and with different disorders. The settings from which telepractice was conducted was diverse: private office, rehabilitation center, special education, school, and hospital.

Satisfaction with telepractice was high among both SLPs (81%) and clients (89%). Being able to continue the speech therapy in safe health conditions (during the COVID-19 pandemic) was an important motivation. Both for SLPs and their clients, satisfaction was related to the fact that telepractice was the only way to continue speech therapy during the period of lock-down. Not having to travel was mentioned as an important advantage. Therapy delivered via telepractice was reported by SLPs to be more structured, an advantage of telepractice. Clients reported that telepractice increased their motivation to participate in therapy sessions.

Logically, these benefits only apply when the telepractice equipment is functioning properly. This requires the availability of a computer or tablet with a well-functioning camera and sound, and a stable and sufficiently fast internet connection. Technical access was generally easy for both the SLPs (84%) and the clients (69%). Among the problems reported, an insufficiently stable internet connection was the most frequently mentioned (86%), along with problems with sound (50%) or image (23%). For more than half of the respondents, problems occurred rarely or occasionally. The ability to avoid transient or consistent barriers for participation and respect for the client's privacy were cited as prerequisites to telepractice.

Some aspects of telepractice were reported by clients as a disadvantage. Mainly, these were problems with equipment, software, and internet. Some clients reported they felt a lack of human contact in telepractice as compared to in-person therapy. The difficulty of specific interventions was also noted (e.g., manipulation of articulators, tongue strength training, role playing, etc.). Another important concern was the need for more concentration and attentional effort when using telepractice. Related to this dissatisfaction were the combined stressors of online schoolwork for the child (36%) and parental telework at home (26%).

Some of the disadvantages reported by SLPs were similar to those reported by clients. Both noted the limited interaction possibilities and the increased effort due to the focus on the screen. More specifically, SLPs mentioned they required more time and effort to prepare telepractice sessions as compared to in-person therapy. In addition, some SLPs noted reduced flexibility to use and switch materials during the telesession.

It was difficult to make exact comparisons of the results of the current study with the earlier described results obtained by Fong et al. (2021), Vrinda and Reni (2020), and Tenforde et al. (2020). First, the survey questions differed among the studies. Second, in the current Belgium study, interventions such as consultation, screening, and diagnosis were not permitted; only the continuation of therapy was allowed.

In general, some client characteristics who participated in telepractice were similar between the studies, such as their wide age distribution. Notable among these studies were the different disorders for which clients are treated by means of telepractice (e.g., articulation and language disorders, speech motor disorders, stuttering, voice disorders, aphasia, dyslexia, etc.). Tenforde et al. (2020) included neurogenic communication disorders.

Telepractice was used in different settings and the proportion of each setting varied across studies. In the current study, private practice was represented more than in the Fong et al. (2021) and Vrinda and Reni (2020) studies. The frequency of use of telepractice could be compared between Fong et al. (2021) and the current study. Fong et al. (2021) reported a less frequent weekly use than was the case for the Belgian SLPs. It is noteworthy that in the study of Fong et al. (2021) and of Vrina and Reni (2020) about three quarters of the SLPs had up to three months experience with telepractice before they were surveyed. In the current study, the maximum was five months of experience before the survey.

Problems with hardware and software were reported by half the participants in the study by Fong et al. (2021), by about one-fourth of those surveyed in the Vrinda and Reni (2020) study and in only 12% in the current study. This was a lesser problem in our study because internet connectivity was predominantly achieved through a cable network.

For estimating satisfaction with telepractice, the studies of Fong et al. (2021), Tenforde et al. (2020), and our study used a different semantic differential. If at least “good” or “satisfied” is considered, Fong et al. (2021) reported 59.7%, Tenforde et al. (2020) 83%, and for the current study 89%.

## CONCLUSIONS AND LIMITATIONS

In the current study SLPs and clients were required to use telepractice to minimize the health risks from infection by COVID-19. The representation of certain age categories and client disorders reflected, among other things, the services offered and the policy of the NIHDI on speech therapy interventions and reimbursement.

Despite the differences between the compared studies and the current study in terms of location, setting, disorders treated, client ages, numbers of respondents, and number of survey questions, common issues emerged when telepractice was suddenly used during the COVID-19 pandemic. Three major “take-aways” are as follows:

1. It is clear that telepractice can be useful for a wide range of disorders and for different age groups and types of clients.
2. There are common barriers that can make telepractice difficult or impossible. These are chiefly technological issues (i.e., computer with camera, image, sound, internet) or user issues (i.e., circumstances, another person helping).
3. There is the future expectation that telepractice can be used outside of a pandemic. However, this requires the provision of education, training, and materials, and recommendations from SLPs who used telepractice, and those who did not.

As limitations, the survey studies considered herein do not allow for statements about measurable effectiveness in terms of achieved modification of a disorder. That requires research with comparative studies between in-person speech therapy and telepractice.

## APPENDIX

### QUESTIONNAIRE USED IN THE VVL-TELEPRACTICE SURVEY

1. Have you used telepractice
  ∘ Yes (→ questions 6 - 38)
  ∘ No (→ questions 2 - 4)
2. If not, why not?
  ∘ I am not trained to give telepractice
  ∘ It requires too much effort to set up telepractice
  ∘ There are technical problems that make telepractice impossible I'm not prepared enough to give telepractice
  ∘ Too few clients qualify for telepractice
  ∘ Others? Please specify.
3. Would you consider doing telepractice in the future?
  ∘ Yes, definitely
  ∘ I might. I am not sure
  ∘ No, definitely not
4. What is necessary for you to do with telepractice? In what way do you think you use it?
5. What did you do to switch to telepractice?
  ∘ I quickly learned how to use the software application.
  ∘ I have used the information from VVL on how to use telepractice.
  ∘ I have received training on telepractice. I
  ∘ I have used material made available in the DIGICENTER of VVL.
  ∘ I have had access to technological support.
  ∘ I already had experience of working online.
6. How many online sessions ref. 30 minutes on average weekly in the last 4 weeks?
  ∘ less than 10 per week on average
  ∘ 11-20 per week
  ∘ 21-30 per week
  ∘ 31-40 per week
  ∘ more than 40 per week
7. How many in-person sessions ref. 30 minutes did you give on average weekly in the last 4 weeks?
  ∘ less than 10 per week
  ∘ 11-20 per week
  ∘ 21-30 per week
  ∘ 31-40 per week more than 40 per week
8. Do you give more or less telepractice NOW than during the lock-down period?
  ∘ less (→ question 9)
  ∘ more ((→ question 10)
  ∘ approximately the same
9. Why do you give less telepractice now than during the lock-down period?
10. Why do you give more telepractice now than during the lock-down period?
11. For which disorders have you used telepractice?
  ∘ aphasia
  ∘ articulation disorders
  ∘ cluttering
  ∘ dysarthria
  ∘ dyscalculia
  ∘ dysphagia
  ∘ dysphasia
  ∘ dyslexia
  ∘ dysortographia
  ∘ dyspraxia
  ∘ hearing disorders
  ∘ oro-myofunctional disorders
  ∘ stuttering
  ∘ voice disorders
  ∘ language development disorder
  ∘ Others? Please specify.
12. How did you perform telepractice in speech therapy disorders with the following co-morbidities?
  ∘ 1.only in-person
  ∘ 2. predominantly in-person
  ∘ 3. telepractice and in-person
  ∘ 4. predominantly telepractice
  ∘ 5. only telepractice
  ∘ 6. not applicable - I did not treat this disorder lately
13. With which age groups of clients could you use telepractice? Toddlers (2–6 years)
  ∘ Children (7-12 years)
  ∘ Teenagers (13-17 years)
  ∘ Adults (18+ years)
  ∘ Parents for parental guidance
14. Are there certain clients or target groups of clients who use telepractice more than others? If so, which? Why?
15. In which setting do you offer telepractice?
  ∘ Private practice □1.Yes □2. No □3 I do not work in this setting
  ∘ Hospital □1.Yes □2. No □3 I do not work in this setting
  ∘ Special education □1.Yes □2. No □3 I do not work in this setting
  ∘ School □1.Yes □2. No □3 I do not work in this setting
  ∘ Rehabilitation center □1. Yes □2. No □3 I do not work in this setting
  ∘ Other? Please specify.
16. Which hardware do you use to offer telepractice?
  ∘ Desktop computer
  ∘ Portable laptop computer
  ∘ Smartphone
  ∘ Tablet
17. Which operating system do you use for telepractice?
  ∘ Linux
  ∘ Mac OS
  ∘ Windows
  ∘ Another? Please specify.
18. Which software application do you use to do telepractice?
  If you used several, please rank them in order of importance. The most important being 1, then 2, etc.
  ∘ FaceTime □1 □2 □3 □4 □5 □6 □7 □8
  ∘ Jitsi Meet. □1 □2 □3 □4 □5 □6 □7 □8
  ∘ Skype □1 □2 □3 □4 □5 □6 □7 □8
  ∘ Skype for Companies □1 □2 □3 □4 □5 □6 □7 □8
  ∘ Teams □1 □2 □3 □4 □5 □6 □7 □8
  ∘ Vectra □1 □2 □3 □4 □5 □6 □7 □8
  ∘ Zoom □1 □2 □3 □4 □5 □6 □7 □8
  ∘ Another? Please specify □1 □2 □3 □4 □5 □6 □7 □8
19. Have you experienced any problems with telepractice? Yes rather often
  ∘ Yes occasionally
  ∘ Yes sometimes
  ∘ Yes rather rarely
  ∘ No
20. If you had problems with telepractice what kind of problems?
  ∘ with the internet connection
  ∘ with the sound (distortion, echo)
  ∘ with the visual display (sharpness, lighting)
  ∘ with the operation of the software application
  ∘ with certain therapy exercises
  ∘ with the digital reproduction of material
  ∘ with the interactions between speech-language therapist and client
  ∘ because of insufficient privacy
  ∘ because of the circumstances (noisy)
21. How satisfied are you in general with the provision of telepractice?
  ∘ 1: Very dissatisfied
  ∘ 2: Dissatisfied
  ∘ 3: Rather dissatisfied
  ∘ 4. Neutral
  ∘ 5: Rather satisfied
  ∘ 6. Satisfied
  ∘ 7: very satisfied
22. In general, how would you rate the satisfaction of the clients who received telepractice?
  ∘ 1: Very dissatisfied
  ∘ 2: Dissatisfied
  ∘ 3: Rather dissatisfied
  ∘ 4. Neutral
  ∘ 5: Rather satisfied
  ∘ 6. Satisfied
  ∘ 7: Very satisfied
23. In your opinion, what makes clients experience telepractice favorably?
  ∘ It was the only way to receive speech therapy care
  ∘ No travel to the practice needed
  ∘ Telepractice could be practiced as efficiently as face-to-face therapy
  ∘ The video technology software used could be easily operated (e.g., sound, picture)
  ∘ A good internet connection
  ∘ Other? Please specify
24. What makes clients experience telepractice unfavorably?
  ∘ There were problems with the computer and/or the internet connection.
  ∘ There was no interest in telepractice *a priori*.
  ∘ The therapy care which was not possible in-person was simply postponed or suspended.
  ∘ The combination with online schoolwork made telepractice impossible because that was too demanding.
  ∘ Parents working at home made telepractice impossible because it was too much of a burden.
  ∘ Client could not sit alone in a room to receive telepractice.
  ∘ Other, please specify.
25. Give advantages or benefits of telepractice for you as a speech-language pathologist.
26. Please give disadvantages or drawbacks of telepractice for you as a speech-language pathologist.
27. Give advantages or benefits of telepractice for your clients.
28. Please list disadvantages or drawbacks of telepractice for your clients.
29. What is your experience of time spent preparing with telepractice?
  ∘ The preparation takes more time for telepractice than for in-person therapy.
  ∘ The preparation is similar to in-person therapy.
  ∘ Preparation takes less time with telepractice than with in-person therapy.
  ∘ Other? Please specify.
30. What is your experience in terms of time spent in succession of sessions with telepractice?
  ∘ The telepractice sessions cannot be held consecutively.
  ∘ The telepractice sessions can be held consecutively.
  ∘ Otherwise? Please specify.
31. What is your experience of the effort involved in using telepractice?
  ∘ The experienced effort is greater with telepractice than with in-person therapy.
  ∘ The experienced effort with telepractice is equal to that with in-person therapy.
  ∘ The experienced effort with telepractice is less than that with in-person therapy.
  ∘ Otherwise? Please specify.
32. What were the costs of telepractice? (i.e., investment in hardware or software, materials, and time)
  ∘ The costs of telepractice and in-person therapy are almost the same.
  ∘ Relatively speaking telepractice costs more than in-person therapy.
  ∘ Relatively speaking telepractice costs less than in-person therapy.
33. How do you experience the accessibility of telepractice for you as a speech-language pathologist?
  ∘ Smooth, little, or no problems
  ∘ Problems creating and using the link of the software for telepractice
  ∘ Problems with logging in for the speech-language pathologist
  ∘ Problems with the operation of the software application by the speech-language pathologist
  ∘ Other, please specify
34. How do you experience the accessibility to telepractice for your clients in general?
  ∘ Generally smooth, few or no problems
  ∘ Problems with using the link of the telepractice software
  ∘ Problems with logging in for the client
  ∘ Problems with the operation of the software application by the client.
  ∘ Other, please specify
35. Will you continue to use telepractice in the future (even after the COVID period)?
  ∘ Yes, surely.
  ∘ Possibly
  ∘ No, definitely not.
36. If you will use telepractice in the future, with which age groups of clients?
  ∘ Toddlers (2-6 years)
  ∘ Children (7-12 years)
  ∘ Teenagers (13-18 years)
  ∘ Adults (18+ years)
  ∘ Parents for parental guidance
  ∘ Discussions (e.g. with teacher)
37. If you are going to use telepractice in the future for which kind of disorders?
  ∘ aphasia
  ∘ articulation disorders
  ∘ cluttering
  ∘ dysarthria
  ∘ dyscalculia
  ∘ dysphagia
  ∘ dysphasia
  ∘ dyslexia
  ∘ dysortographia
  ∘ dyspraxia
  ∘ hearing disorders
  ∘ oro-myofunctional disorders
  ∘ stuttering
  ∘ voice disorders
  ∘ language development disorder
  ∘ Others? Please specify.
38. Comments, remarks, suggestions regarding telepractice.
